# Women's Care in First‐Episode Psychosis: Clinicians' Perspectives on Service Provision

**DOI:** 10.1111/eip.70163

**Published:** 2026-04-09

**Authors:** Grainne McGinty, Sean Naughton, Keith Gaynor, Saoirse Byrne, Roisin Farrelly, Anja Stano, Mary Clarke

**Affiliations:** ^1^ DETECT Early Intervention in Psychosis Service Dublin Ireland; ^2^ Saint John of Gods Research Foundation, Psychosis Research Centre Dublin Ireland; ^3^ School of Medicine, University College Dublin Dublin Ireland; ^4^ School of Psychology, University College Dublin Dublin Ireland; ^5^ Max Plack Institute for Human Development Berlin Germany

**Keywords:** early intervention in psychosis, first episode psychosis, sex differences, womens mental health

## Abstract

**Background:**

Early intervention in Psychosis (EIP) services are vital in reducing the long‐term impact of first‐episode psychosis (FEP). However, women with FEP encounter unique biological, social, and systemic challenges that may delay diagnosis, hinder access to care, and affect treatment engagement.

**Aims:**

This study explores clinicians' perspectives on the sex‐specific needs of women with FEP, focusing on hormonal influences, caregiving responsibilities, stigma, and systemic barriers to care within EIP services.

**Method:**

A qualitative design was employed, utilising semi‐structured interviews with 20 clinicians from EIP and adult mental health services in Dublin, Ireland. Reflective Thematic Analysis was used to identify key themes, with an inductive constructivist approach underpinning the analysis.

**Results:**

Four key themes: (1) Sex differences in presentation, with women showing later onset, higher emotional distress, and lower rates of substance‐induced psychosis; (2) Hormonal interactions, highlighting the impact of menstruation, pregnancy, and menopause on symptomatology and treatment response; (3) Shame and adjustment, with internalised stigma, delayed help‐seeking, and disruption of social roles; (4) Barriers to care, including caregiving responsibilities and inflexible service models. Clinicians emphasised the need for integrated, sex‐sensitive and trauma‐informed care, as well as improved collaboration between psychiatry, gynaecology and endocrinology.

**Conclusions:**

Women with FEP face multifaceted barriers to accessing and engaging with EIP services. Implementing clinician recommendations, namely flexible and integrated care, trauma‐informed and sex‐sensitive approaches, and robust cross‐disciplinary collaboration, is vital for improving outcomes. Future research should incorporate service user perspectives to further refine inclusive and effective EIP pathways for women.

## 1 Introduction

Early Intervention in Psychosis (EIP) services play a crucial role in reducing the long‐term impact of first‐episode psychosis (FEP). Yet women with FEP face distinct challenges that can delay diagnosis, limit access, and hinder engagement (Mazza et al. 2021). Although sex and gender differences in psychosis are increasingly recognised, important gaps remain in understanding how biological, social, and structural factors shape women's experiences (Barker and Vigod 2023). Our analysis examines how clinicians respond to women's needs within EIP services, adopting a biosocial perspective that acknowledges the interaction of sex‐linked hormonal processes with gendered social roles. While the study focuses on cisgender women, we recognise that sex and gender are non‐binary constructs that intersect in complex ways.

Compared with men, women tend to experience a later onset of FEP, typically in their late 30s to mid‐40s. This pattern is linked to sex‐specific hormonal transitions, particularly fluctuations in oestrogen during perimenopause and menopause, which may influence neural pathways implicated in psychosis (Häfner et al. 1994; Häfner and an der Heiden 1997; Seeman 1996; Riecher‐Rössler and Häfner 2000; Cotton et al. 2009; Ochoa et al. 2012; Jongsma et al. 2019). Middle‐aged women are disproportionately represented in adult EIP services: in a London cohort, 32.5% of service users were over 35, with women overrepresented (Clay et al. 2018), while Fernandes et al. (2022) found that 61.5% of late‐onset (≥ 40 years) cases were women. Despite this, middle‐aged women remain underrepresented in EIP evaluation and service design. Integrating awareness of hormonal transitions, including menstruation, pregnancy and menopause, within EIP frameworks would strengthen sex‐sensitive care (Ferrara and Srihari 2021). 

Beyond biological factors, social determinants also shape women's experiences of EIP care. Caregiving responsibilities can be a major barrier to accessing treatment, particularly as traditional EIP models often do not accommodate the needs of women with childcare obligations (Ferrari et al. 2018). Although caregiving can involve any gender, women constitute the majority of primary caregivers within psychosis populations (Radley et al. 2022). In one clinical sample of 709 patients, 56% of women had children compared with 30% of men, a pattern consistent across diagnoses (Schrank et al. 2016). Social expectations and stigma surrounding psychosis can further deter women from seeking help, including fears of judgment or concerns about the impact on their children (Diaz‐Caneja and Johnson 2004; Gronholm et al. 2017; Seeman 2023).

Current EIP models, still largely designed around youth‐onset psychosis, often fail to consider the hormonal and psychosocial factors central to many women's experiences of FEP. Meeting these needs requires approaches that actively engage with the social and structural realities shaping women's lives. Taken together, women's later illness onset, caregiving responsibilities and intersecting psychosocial stressors, including stigma, have prompted growing calls in the literature for coordinated, multidimensional models that address both biological and social determinants of mental health (Mazza et al. 2021). Despite extensive evidence on sex differences in psychosis, translation into routine practice remains limited (Naughton et al. 2024). Many women experience FEP during periods of major hormonal change while managing substantial social and caregiving demands, underscoring the need for care models that reflect these overlapping realities. Integrated approaches that bring together psychiatry, endocrinology and gynaecology, similar to established perinatal or reproductive mental health services, offer a potential blueprint for holistic, sex‐ and gender‐responsive EIP care. Although examples of such integrated psychiatric–reproductive models demonstrate feasibility, they remain rare within EIP services (González‐Rodríguez et al. 2023). This study therefore examines clinicians' perspectives within an Irish EIP network serving adults aged 18–65 to inform the development of more flexible, inclusive and sex‐ and gender‐responsive approaches to early psychosis care.

## 2 Methodology

### 2.1 Design and Ethics

This qualitative study used semi‐structured interviews with clinicians working in an EIP and adult mental health service in Dublin. It forms part of a larger project that also includes women's perspectives and quantitative analyses of their presentations and outcomes. An inductive, constructivist approach underpinned the study, recognising knowledge as co‐constructed through participants' experiences and contexts. Reflexive thematic analysis (RTA; Braun and Clarke 2021) enabled interpretative engagement with the data. RTA was chosen because sex‐ and gender‐responsive practice in FEP is underexplored and a reflexive design allowed unanticipated insights into the biosocial dimensions of care. Four themes were constructed through RTA via iterative coding, refinement, and critical reflexive engagement with the dataset by the researchers.

The topic guide, developed following a literature review, explored clinicians' views on women's needs in FEP care and perceived gaps in current service provision (Please see Table 1). Guided by a constructivist epistemology, the study examined how clinicians make sense of sex and gender in their clinical roles, and how these meanings shape engagement, care priorities, and team dynamics. Interviews were treated as co‐constructed exchanges, with the researcher and participant jointly shaping meaning. Reflexive diary notes captured these interactions and informed both questioning and interpretation.

**TABLE 1:** Topic guide for qualitative interviews with clinicians.

Topics	Questions
Topic 1: Differences in presentation and care needs	1.1: Have you noticed any differences in how women present with a First Episode Psychosis (FEP)? For instance, do you think there might be variations in the onset period and early symptoms? 1.2: What's your take on whether women with FEP have specific care needs? Are there any examples you can think of that illustrate these differences?
Topic 2: Pathways to care and engagement with mental health services.	2.1: Do you think there are differences between men and women in how they seek help from mental health services when experiencing a FEP? Are there any obstacles or hurdles that you believe are more specific to women? 2.2: How do you feel about considering hormone cycles in the care pathways and outcomes for clients with FEP? Do you think it could make a difference? 2.3: In your experience, are there particular challenges in keeping women engaged in mental health services after their initial contact? At what points in the care process do you usually see these challenges arise?
Topic 3: Care planning and delivery for women with a FEP	3.1: When planning care for women with FEP, do you routinely take into account any sex‐specific issues? For example, how do you address psychological support, social and occupational planning, physical health monitoring, or prescribing medication? 3.2: Have you observed any differences in clinical outcomes between men and women with FEP? If so, what do you think might mediate these differences, whether it's in symptomatic outcomes or functional outcomes like social, occupational, or economic aspects? 3.3: From your perspective, what kinds of improvements or additional supports could be put in place to better address the specific needs of women with FEP? And why do you think these changes would be beneficial? Have you come across any successful examples of such supports being implemented?

Ethical approval was granted by the St John of God Research Ethics Committee (ID2024‐2037), and procedures adhered to the 1964 Declaration of Helsinki and its later amendments.

### 2.2 Participants and Procedure

Clinicians were recruited from St. John of God Community Mental Health Services, Dublin (catchment ~165 000), including the Dublin East Early Treatment and Care Team (DETECT) and Cluain Mhuire, a secondary mental health service, which accepts referrals from primary care, hospitals, and other community teams. A purposive sampling strategy, incorporating elements of maximum variation sampling, was used to ensure diverse and representative perspectives. Participants were selected based on professional roles and relevance to the study aims, drawing from DETECT, the crisis resolution team, and general adult CMHTs.

Twenty clinicians participated, including psychiatrists, psychologists, mental health nurses, social workers and occupational therapists (Please see Table 2). Participants were not asked to categorise patients by gender identity, and clinicians generally spoke in binary, cis‐normative terms. Participants were approached via a gatekeeper, provided with study information, and contacted by the research team if agreeable. Recruitment spanned 9 months and continued until thematic sufficiency was reached, ensuring both depth of interpretation and diversity of clinician perspectives across service settings.

**TABLE 2:** Participant demographics.

Profession	*N* (%)
Clinicians	20 (100)
Psychiatrists	5 (25)
Senior clinical psychologists	3 (15)
Mental health nurses	8 (40)
Social workers	2 (10)
Occupational therapist	1 (5)
Key worker	1 (5)
Sex	
Male	7 (35)
Female	12 (60)
Age	
25–34	0 (0)
35–44	9 (45)
45–54	5 (25)
55–65	7 (35)
65+	0 (0)

### 2.3 Data Analysis

Interview data were transcribed and analysed using RTA, emphasising researcher reflexivity and iterative engagement (Braun and Clarke 2021). Data familiarisation involved repeated transcript readings, note‐taking, and reflexive journaling to record first impressions and contextual observations. Initial coding was conducted in Microsoft Excel and then refined into thematic categories and final themes. Participant quotes support interpretations, with anonymised identifiers indicating professional roles.

#### 2.3.1 Researcher Reflexivity

Coding was conducted collaboratively by two researchers (G.M. and S.B.), with regular discussions to refine themes and challenge assumptions. Reflexive memos documented interpretative decisions and researcher positioning throughout. Field notes and diaries were reviewed during theme development to enhance transparency and maintain consistency with the constructivist stance. Reflexive discussions also addressed the research team's assumptions about sex and gender, recognising that clinicians' accounts, and our interpretations, were shaped by prevailing binary frameworks within mental health services. A detailed reflexivity statement is provided in Supporting Information.

## 3 Results

**FIGURE 1:**
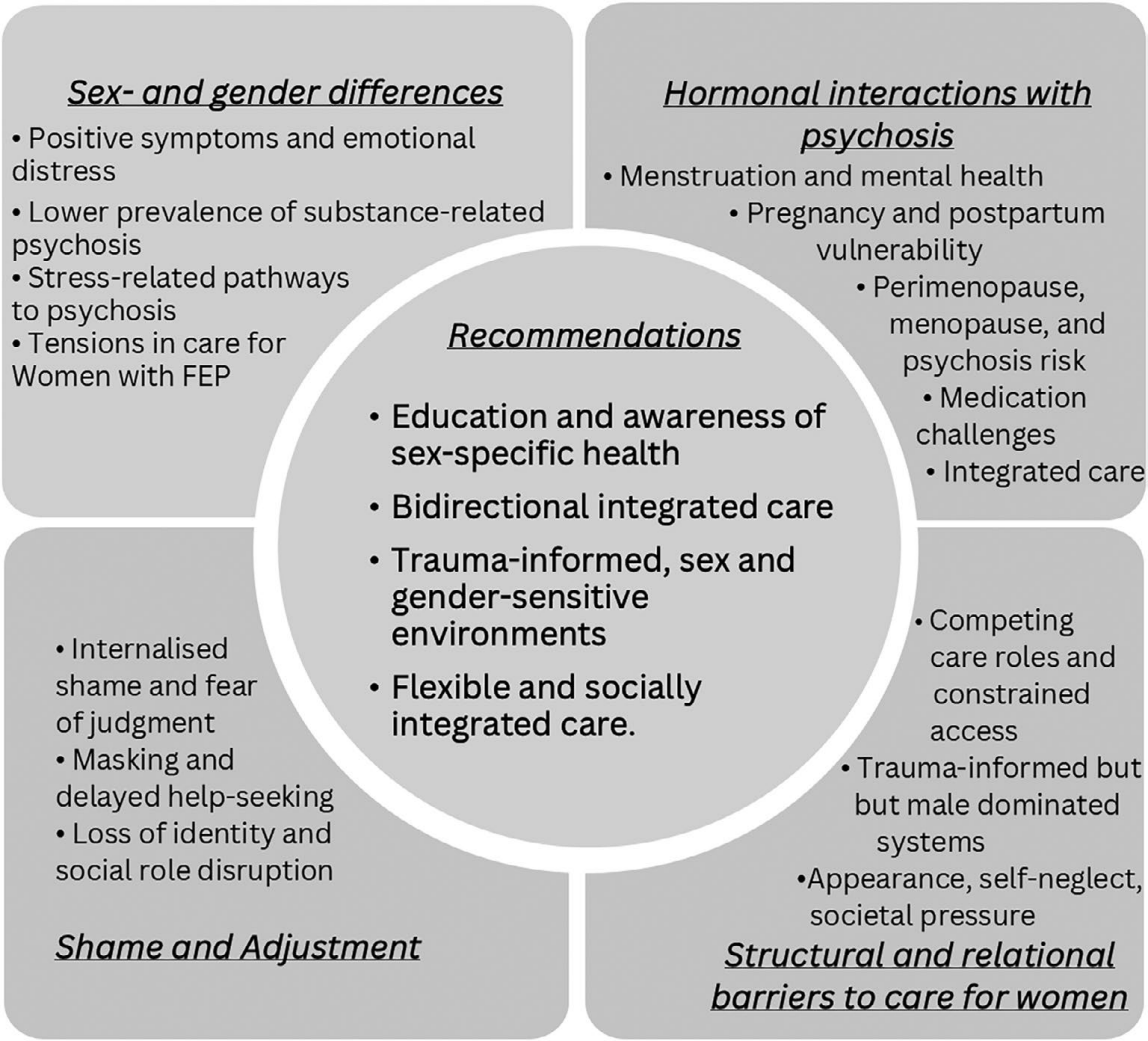
Themes and recommendations from clinicians interviews regarding women's FEP care.

Thematic analysis identified four key themes: (i) sex and gender differences in presentation; (ii) hormonal interactions with psychosis; (iii) shame and adjustment; and (iv) structural and relational barriers to care for women (see Table 3 for supporting quotes). Please see Figure 1 for themes.

**TABLE 3:** Themes and recommendations from clinicians' interviews.

Theme	Subthemes	Quote
1. Sex differences in presentation	Established sex differences	Maybe the past few years is that there be more of a late onset presentation for women, you know, with no family history of mental illness or no history themselves of any mental health conditions, who were in their 40s and 50s, they they're presenting to the service with for first episode psychosis. Transcript 1
		I think there are lots of differences ‘ehm’ I think I think women, I mean the the one, the first thing is that my in my experience with with with women is that they tend to be much older… (Speaker 1: Yeah) ‘ehm’ so a lot of women kind of in their 40s, early 50s… ‘ehm’ sadly—Transcript 9
		I would also say, for the most part, not completely, but for the most part. It is not substance related. (Speaker 1: Yeah). Whereas, in relation to men you know we get … we're nearly questioning if the substance isn't part of, (Speaker 1: Yeah)—Transcript 6
	Stress‐related pathways to psychosis	I think in FEP literature that emotional regulation difficulties…can predate now, they're not necessarily predictable, but can predate a young person presenting in that bracket the 18‐ to 25‐year‐old bracket. But I also think similarly for women postnatally, there's a quite an instance of, I think, ‘um’, emotional regulation difficulties and they are of course, presenting a good bit later than your usual cohort of 18/25—Transcript 12
		So the women that I have assessed. Through detect all, bar one, we're experiencing. Stress. And some unaddressed trauma had resurfaced—Transcript 17
		As a group, there may be an increased tendency for the psychosis to present with positive psychotic symptoms as a primary presenting feature, so delusions and hallucinations I feel are more prevalent than maybe the negative symptoms which can affect social and occupational functioning and. And I also think that there's an increased prevalence of sudden onset presentations where there might be a sudden change in functioning that leads to an early presentation just touching services—Transcript 3
	Tensions in care for women with FEP	‘Ehm’ so differences in their presentation, probably not hugely to be honest I mean, I suppose the ones I have more worked with have been those who have had kind of more negative symptoms. So kind of more withdrawn or flattened ‘ehm’…‘ehm’. Yeah, and that would be similar to those I've worked with like in the male population—Transcript 11
		They're much less likely to seek help much more suspicious (Speaker 1: Hmm)—Transcript 9
		I mean I I think the prognosis for a man is much better than for a woman, ‘Ehm’…and I don't know whether that's about the later presentation and that there's…there's more social capital lost (Speaker 1: Okay) for a woman you know, or whether it's just then there isn't that opportunity to intervene they will have suffered in silence for a lot longer—Transcript 9
		Yeah. I'd say probably I think with females, if they have a lot of support … or they, they kind of take on the support and they're encouraged to kind, of stick with. Their outcomes improve, significantly. Like I'd say, what with given a lot of encouragement and support that happens—Transcript 5
		We tend to get that the women tend to follow through. More in relation to. Following up, they might be more conscientious about seeing the process through—Transcript 6
		So…no, is the short answer. There may be an age difference, but not one that jumps out massively—Transcript 12
2. Hormonal interactions with mental health	Menstruation and mental health	I don't know if it's true or not, but definitely for some women, mood disorders such as schizoaffective disorder may be associated with menstruation, with variation of their mood plus or minus psychotic symptoms—Transcript 12
		Menstrual cycles and perimenopausal periods are not often considered in clinical practice, and evidence on their impact on psychosis is limited—Transcript 3
		I think it's it can be really frustrating for me that even the younger clients I see that have really kind of painful irregular periods, constant bleeding, things like that. I'm, you know, anecdotally thinking there must be something going on here—Transcript 7
	Pregnancy and postpartum vulnerability	It's more the post‐natal depression and post‐natal psychosis. Women with post‐natal psychosis have over double the risk of relapse in a second pregnancy—Transcript 2
		I've worked in perinatal psychiatry, and women who present for the first time following childbirth… it is the highest risk time in a woman's life for any mental health problem—Transcript 12
		It's something that women are questioning more—could it be menopause playing a role?—Transcript 14
		I would not be shocked if an increased incidence of psychosis around the onset of menopause was found—Transcript 14
	Perimenopause and menopause	The lack of research in perimenopause is evident. You start hormonal replacement treatment, but there are no follow‐ups, and you don't know if higher doses could induce psychosis—Transcript 2
		Perimenopausal and postmenopausal periods are associated with a higher prevalence of mental health conditions, yet this is not often considered when treating psychosis—Transcript 3
		The protective effect of being premenopausal may be lost after menopause, potentially affecting recovery rates—Transcript 3
	Medication challenges and hormonal fluctuations	Prescribing meds …‘eh’ I would if, so depending on, you know, whether someone is pregnant, planning on being pregnant that kind of thing absolutely …‘eh’ sex specific … I do try to avoid prolactin, you know, ones that cause high prolactin tend to avoid them—Transcript 12
		For a young woman who's not maybe in a relationship, or not really thinking I I think the responsibility is ours as prescribers in the community mental health teams to be having that conversation, (Speaker 1: Yeah) ‘ehm’ with women. And there isn't a lot of information readily available—Transcript 12
		‘um’ and then you have the whole issue are we damaging someone's fertility?—Transcript 14
	The need for integrated care	There's such a lack of joined‐up thinking in healthcare—Transcript 7
		More awareness and education are needed around menopause, perimenopause, and menstrual cycles in relation to mental health—Transcript 7
3. Shame and adjustment	Internalised shame and fear of judgement	Much more ashamed ‘ehm’ than than men, so that so that those are the factors, so I don't know if the age of onset, I don't know, that's something to do either socially or psychologically or whether there's a biological thing going on. ‘Ehm’ but also they seek help differently—Transcript 9
		Because, there is very, even in terms of menopause, there is a lot of stigma and, women are going through all those second changes in life, and there is not a lot of supports and there is still not open dialogue about what happens on menopause and perimenopause—Transcript 2
		I I just feel like there's there's…that that shame seems to be a lot more prominent—Transcript 7
	Masking	I suspect that women may be…whether it's true socialisation or culture or whatever it might be may be better at masking what is going on for them, em, so that others aren't noticing as quickly as they might with with somebody else. And it also may be that, with men, symptoms may be more dramatic or obvious, em, where they may be much more subtle. Em, or kind of, reserved is not the right word, but just sort of. Dampened down a little bit, by women, it's just not quite as noticeable—Transcript 6
	Loss of identity and social role disruption	It's even the value of a woman is very often still self‐determined not even by other people, but ‘ehm’ very often by connectedness and the ability to care for others and all that is blown out of the water in psychosis—Transcript 14
		If you're working at a certain age with a family and everything like that, and a job it can be hard to like, to meet up with your friends anyway, (Speaker 1: Yeah) but you know, if you if you have a diagnosis of of FEP ‘ehm’…and you don't really feel like you're engaging in all of your roles as well as you used to then that can also serve to be really isolating for someone—Transcript 10
4. Structural and relational barriers to care for women	Competing care roles and constrained access	Speaker 1: What kind of barriers would would stick out then for… Speaker 2: Well the childcare is is a big one—Transcript 9
		I suppose the other… big one (barrier) that's always been there is that the biggest barrier for a lot of women coming to interventions is childcare—Transcript 6
	Trauma‐informed but male‐dominated systems	I think trauma could be put as a precursor to ‘um’ to psychosis and many other forms of mental illness. (Speaker 1: ‘Mhm’) And very often it's trauma being perpetuated by men and very often these women come into services that are heavily staffed by men and their less their less likely to engage and even if they do engage it's more superficial and less forthcoming.—Transcript 14
		Childhood adversity is a one of the key markers. So I think that maybe. Trauma, you know, in terms of a risk factor, there's probably more—Transcript 1
		I think particularly in female patients where there may be a history of trauma. And I would be keen to assist. Take that into account when deciding on a on a therapist that could see the patient—Transcript 3
		I would have worked with a number of women who'd been sexually assaulted in while impatient ‘um’ and, you know, how, when you're so traumatised already and then that occurs when you're psychotic and the ability to make sense of that afterwards is so… it's so confusing—Transcript 11
		Well, again like the fact that a lot of it it like it's later in life because a lot of this trauma related, maybe there needs to be more of an emphasis for psychological support for women. And kind of find out the root causes as well and kind of get support around that. And then there might be a need for maybe additional family support if they there is a family there, there are children—Transcript 19
	Appearance, self‐neglect, and social pressure	For women…’ehm’…and then the other bit is the medications then that we prescribe, there they often weight gain and it's such a big barrier for women, I mean in terms of compliance its huge, its huge yeah (Speaker 1: Okay) yeah in terms of what I've seen over the course of my career, just that's the main they just really struggle with that, (Speaker 1: ‘Umm’) yeah, really difficult. (Speaker 1: Yeah). As men… they struggle, but not the the body image stuff isn't just isn't as prominent. Yeah—Transcript 9
		It's great in the acute phase but in the longer term phase it can lead to metabolic syndrome and obviously that's bad for everyone but it is even worse and less palatable for women who…may be able to turn look away from the metabolic syndrome but their not able to look away from the weight gain and societal ‘um’… sidelining that happens as a result of that so, you know, despite body positivity and what not the reality is weight matters a lot, to women—Transcript 14
		I think there's probably more competing demands and I wonder…like, again like with the say with the younger guys that I'm working with, a lot of them are still living at home and they they have less responsibilities I suppose. Whereas with the women…they, there is more expected of them—Transcript 10
		I think also that, you know the idea of talking about yourself and your own right… for a lot of women can be…socially, you know, unfamiliar—Transcript 9 I think and I suppose maybe…maybe I suppose the stigma around medication and weight gain puts certain women off—Transcript 14
5. Recommendations for improving care for women	Enhanced education and training	I think that's something I was never really acutely aware of until I started working in the field. I don't think it's anything that from what I can recall anyway that was ever taught or read about the textbooks or even at any stage sort of almost acknowledged throughout the earlier parts of my training, But it's kind of something as I've progressed through training, I've progressed through the years that I've become sort of a little bit more aware of. (Transcript 18)
		Education. In terms of the professionals, we need to be more aware about hormonal changes and risks associated with them—Transcript 2
	Bidirectional integrated care	There needs to be more links between gynaecology and endocrinology and psychiatry—Transcript 7
		Those are links I suppose I'd like to see being a lot stronger between community mental health teams and the community—Transcript 12
	Trauma‐informed, sex and gender‐sensitive environments	Women‐only clinics, consistent medics, the option for a female therapist, women‐only groups, and spaces in inpatient units. Transcript 7
		Being mindful of the contribution that trauma may have played, female sexual trauma in particular, prior to becoming psychotic. Transcript 14
	Flexible and socially integrated care	Interestingly, that family were very angry about the illness … that happens with women who are mothers, the family gets very angry that the mum is unwell—Transcript 12
		More support for their families, so people can do the work—Transcript 7
		I'd agree with the suggestion that any additional childcare supports that could be provided to this population would be beneficial—Transcript 3 It's something I've thought about probably more on a staffing level … child support and help with childcare, whether there's funding or linking in with existing childcare services, creches or whatever—Transcript 4

### 3.1 Theme 1: Sex‐ and Gender‐Related Differences in Presentation

#### 3.1.1 Established Sex Differences

Clinicians consistently identified well‐recognised differences: women were perceived as presenting later in life, with fewer substance‐related psychoses and a higher prevalence of affect‐laden positive symptoms.

There are fewer substances involved and their psychoses tend to be more organic … delusions tend to be less dramatic and concerned with their domestic situation. (Psychiatrist, T14).

Mostly you see women at middle age. (Nurse, T7).

The onset tends to be more late in life, particularly with perimenopause and post‐menopause. (Psychiatrist, T2).

Men's psychoses are often drug‐induced … that hasn't been the case for women. (Psychologist, T6).

These differences were largely taken as familiar clinical knowledge. Several clinicians linked later onset to accumulated life experience, social integration, and established caregiving roles that shaped how symptoms were expressed, recognised, and managed in services.

### 3.2 Stress‐Related Pathways to Psychosis

Clinicians' narratives reflected both biological (e.g., hormonal) and psychosocial (e.g., caregiving, relational stress) factors, underscoring the biosocial nature of women's psychosis pathways. Many conceptualised women's psychosis as emerging through emotional or stress‐related pathways characterised by anxiety and distress:

As a group, there may be an increased tendency for psychosis to present with positive symptoms … delusions and hallucinations are more prevalent. (Psychiatrist, T3).

They become overwrought or anxious … and sometimes that will develop into psychosis. (Nurse, T5).

Women who've gotten acutely unwell … often have a history of low‐to‐medium‐grade emotional‐regulation difficulties. (Psychologist, T12).

Some clinicians linked this to hormonal transitions such as perimenopause, while others framed it as a vulnerability shaped by relational and caregiving demands. One clinician observed that some women displayed negative symptoms ‘similar to the male population’ (Psychiatrist, T11), highlighting that these pathways are not uniform.

#### 3.2.1 Tensions in Care for Women With FEP

While most clinicians were confident that women tended to present later and engage more consistently with services, their accounts also revealed tensions in how these patterns were understood and managed:

They attend appointments, but whether they divulge themselves in this environment is anyone's guess. (Occupational Therapist, 14).

Some questioned whether differences extended beyond age: ‘there may be an age difference, but not one that jumps out massively’ (*Psychologist, T12*) while others challenged biological explanations, suggesting that caring responsibilities might delay help‐seeking: ‘it's hard to know if it's hormones or the fact that women delay coming in because they're caring for everyone else’ (*Psychiatrist, T11*).

Clinicians described women as conscientious and ‘*more compliant*’ yet also reflected that later presentation might contribute to poorer prognosis and loss of social capital. These accounts highlight how clinicians negotiate the gap between established evidence and the complex realities of women's experience in EIP care.

### 3.3 Theme 2: Hormonal Interactions With Psychosis

Theme 2 contained 4 subthemes: (i) later age of onset and hormonal triggers; (ii) menstruation, pregnancy and menopause, and psychosis risk; (iii) medication challenges and hormonal fluctuations; (v) the need for integrated care.

#### 3.3.1 Later Age of Onset and Hormonal Triggers

Clinicians consistently noted a later age of onset for FEP in women, often in middle age and particularly during perimenopause:

Age is definitely a later onset… mostly you see women at middle age (Psychiatrist, T7).

I notice a difference, particularly with perimenopause and post‐menopause… the onset tends to be more late in life (Psychiatrist, T2).

#### 3.3.2 Menstruation, Pregnancy, and Menopause

Menstruation was described as exacerbating anxiety, paranoia, and emotional distress:

You think there's definitely a cyclical connection to it… your mental illness deteriorates around the time of their period (Nurse, T5).

Women were often aware of this pattern and reported anticipating deterioration around their period. Pregnancy and the postpartum period were described as times of heightened vulnerability, particularly for psychosis:

The postpartum period is the highest risk time in a woman's life for any mental health problem (Psychiatrist, T12).

Perimenopause and menopause were seen as key phases that intensified symptoms and complicated treatment, with clinicians noting the lack of dedicated research and follow‐up care for women in this age group.

#### 3.3.3 Medication Challenges and Hormonal Fluctuations

Clinicians described prescribing psychotropic medication for women as complicated by hormonal status, fertility and family‐planning concerns. They highlighted prolactin‐raising antipsychotics and teratogenic mood stabilisers as difficult choices for women of childbearing age, noting the need to initiate conversations about contraception, pregnancy risks and sexual side‐effects in the context of limited sex‐specific evidence:

Pharmacotherapy can be complicated for women… the medication potentially impacts their fertility (Psychiatrist, T3).

#### 3.3.4 The Need for Integrated Care

Clinicians emphasised the need for stronger integration between mental health, primary care, and reproductive health services:

There needs to be more links between gynaecology, endocrinology and psychiatry (Psychiatrist, T7).

One clinician described a women's health hub that co‐located psychiatry, women's health, and childcare, suggesting similar models should underpin care for women with psychosis. Some clinicians had begun routinely enquiring about hormonal stage in assessments, but most highlighted the need for education and shared protocols to avoid treating reproductive and psychiatric care as separate silos.

### 3.4 Theme 3: Shame and Adjustment

Theme 3 comprised three subthemes: (i) internalised shame and fear of judgement; (ii) masking and delayed help‐seeking; and (iii) loss of identity and social role disruption.

#### 3.4.1 Internalised Shame and Fear of Judgement

Many women with FEP were described as experiencing significant shame and self‐stigma around their diagnosis:

By the time they come to me, they are utterly self‐critical. So ashamed. They don't really understand their diagnosis (Psychiatrist, T7).

Clinicians also highlighted fears about parenting and child custody:

The fear of, you know, having children taken away and I know of cases where that has happened’. (Psychiatrist, T7).

#### 3.4.2 Masking and Delayed Help‐Seeking

Internalised stigma often contributed to delayed help‐seeking. Women were described as minimising symptoms and ‘suffering in silence’, which prolonged distress and complicated recovery. Addressing this reluctance was seen as crucial to improving early intervention.

#### 3.4.3 Loss of Identity and Social Role Disruption

Psychosis was reported to disrupt women's roles as caregivers, professionals, and friends, with some experiencing a profound loss of identity in these domains. ‘Women can really feel that loss of identity, whether it's as a mother, a carer, or a good friend’ (*Psychologist, T6*).

### 3.5 Theme 4: Structural and Relational Barriers to Care for Women

Theme 4 contained 3 subthemes: (i) competing care roles and constrained access; (ii) trauma‐informed but male‐dominated systems; and (iii) appearance, self‐neglect, and societal pressure.

#### 3.5.1 Competing Care Roles and Constrained Access

Women's caregiving responsibilities were repeatedly cited as restricting engagement in treatment. Service structures were viewed as inflexible and poorly aligned with women's family and work roles:

A lot of our interventions are 9 to 5… waiting for a partner to get home so they can attend isn't an option. (Nurse, T6).

The lack of childcare support and fears around child protection involvement compounded these difficulties:

We're not well set up to meet the needs of women with family responsibilities… they nearly abdicate them altogether. (Social Worker, T9).

The fear of having children taken away… and I know of cases where that has happened. (Psychiatrist, T7).

These tensions often forced women to prioritise family responsibilities over personal recovery, delaying or interrupting engagement with care.

#### 3.5.2 Trauma‐Informed but Male‐Dominated Systems

Clinicians acknowledged that many women entering services had trauma histories, frequently involving men, yet they encountered predominantly male clinical environments.

It can be very, very scary for women… to enter into a medicalised world that's very male‐dominated. (Psychiatrist, T7).

Many of these women come into services heavily staffed by men… they're less likely to engage and even if they do it's more superficial. (Psychologist, T14).

These dynamics were seen as undermining safety and trust, prompting calls for women‐only spaces and trauma‐informed practice.

If there was a women‐only clinic, like mental health clinic, because I think as I say, so many of the women that we see have experiences of sexual abuse or assault or, you know, having been harmed by men… I think it can feel very unsafe to go into a space where they're only, you know, seeing a male registrar and they've got lots of men around them (Psychiatrist, T7).

#### 3.5.3 Appearance, Self‐Neglect, and Societal Pressure

Weight gain from antipsychotic medication was widely recognised as a major barrier to adherence, reflecting the influence of social expectations around femininity and body image.

The medications we prescribe often cause weight gain, and it's such a big barrier for women… in terms of compliance it's huge. (Social Worker, T9).

Not only have I got this illness and you're telling me I'm not rational, but now you're telling me I'm going to gain weight. (Psychologist, T14).

Beyond body image, clinicians described how societal norms of caregiving and endurance led women to deprioritise their own wellbeing:

It's much easier for women to subjugate their own needs than men… and one of the needs that gets subjugated is mental health. (Social Worker, T9).

Barriers to care reflected intertwined social and biological realities, pointing to the need for flexible, trauma‐informed, and sex‐ and gender‐responsive EIP services.

### 3.6 Reflexive Summary

Across all themes, clinicians' perspectives reflected both shared and discipline‐specific understandings of women's experiences. As researchers, we recognised that our interpretive lens, shaped by feminist and clinical experience, also influenced how these narratives were understood and thematised. This reflexive stance informed the analytic process and ensured that the findings foreground how biological and social factors interact in EIP care.

## 4 Discussion

This study explored clinician perspectives on sex‐ and gender‐responsive care for women with FEP, focusing on hormonal influences, symptom presentation, caregiving roles, and systemic barriers. The findings illustrate the interplay between biological processes and gendered social contexts, a biosocial understanding central to advancing sex‐ and gender‐responsive early‐psychosis care.

### 4.1 Sex and Gender Differences in Symptoms and Presentations

Clinicians noted sex‐ and gender‐related differences in presentation, describing women as showing greater emotional distress and lower rates of substance‐related psychosis. These perceptions partly align with meta‐analytic findings showing higher affective symptoms and lower substance misuse among women, while evidence on positive symptoms is mixed and may indicate greater severity in men (Carter et al. 2022). Although research on DUP is inconsistent, some clinicians perceived delayed detection in women, attributing this to a subtle affective prodrome and to early symptoms being masked or normalised. Pathway‐to‐care research similarly suggests that gender stereotypes can delay recognition of psychosis in women (Ferrari et al. 2018). While not universal, these patterns highlight the importance of diagnostic frameworks sensitive to sex and gender differences in early intervention.

### 4.2 Hormonal Transitions and Integrated Care

Hormonal transitions, including menstruation, pregnancy, the postpartum period, and menopause, were consistently described by clinicians as periods of heightened vulnerability yet areas in which they had received limited training or guidance. Perimenopause and menopause, in particular, were linked to relapse or later‐onset psychosis, consistent with evidence that declining oestrogen levels increase susceptibility to psychotic disorders (Seeman 1996; Ferrara et al. 2024). While clinicians in this study worked within an Irish service extending EIP access up to age 65, many international programs restrict eligibility to ages 18–35 (Birchwood et al. 2013; McGorry et al. 2018). Such thresholds risk excluding women whose psychosis emerges during midlife hormonal transitions, underscoring the need to broaden eligibility criteria to reflect sex‐ and life‐stage‐specific vulnerabilities (Fernandes et al. 2022; Clay et al. 2018).

Hormonal fluctuations were also said to complicate pharmacological management, with clinicians reporting reduced medication efficacy and heightened side effects during hormonally active periods, a challenge well documented in reproductive psychiatry (Howard et al. 2025). Participants emphasised the need for closer collaboration between psychiatry, gynaecology, and endocrinology, noting that fragmented referral pathways and limited shared decision‐making often undermine continuity of care. Such coordination was described as inconsistent and largely dependent on individual initiative rather than formalised pathways. Embedding hormonal health specialists within EIP teams was proposed as a means to support real‐time consultation and integrated management, echoing successful approaches used in perinatal and reproductive mental health services (Barker and Vigod 2023; Mazza et al. 2021).

### 4.3 Sex and Gender‐Specific Issues in EIP Service Delivery

Clinicians identified persistent structural and relational barriers limiting women's engagement in EIP. Consistent with previous studies (Caputo et al. 2016; Gronholm et al. 2017). Rigid 9–5 schedules and lack of childcare were seen as major obstacles to engagement with care for women balancing employment and caregiving. One participant referenced suggesting that family‐inclusive principles could inform EIP design, for example, a women's health hub co‐locating psychiatry, primary care, and childcare supports Evidence from integrated women's and family‐health hubs shows that co‐located reproductive, mental, and social supports improve accessibility and continuity (Loveday et al. 2024). Clinicians noted that such models could also ease scheduling pressures, allowing women to attend appointments within working hours knowing their children are cared for. This dual benefit, alongside flexible scheduling and home visits, could improve both service‐user engagement and staff wellbeing (Radley et al. 2022; Redmond et al. 2025).

Shame, stigma and fear of child custody loss were also critical concerns, with clinicians reporting that many women delayed help‐seeking due to stigma. This underscores the importance of addressing societal stigma and raising awareness, so women feel safe seeking help without fear of punitive consequences. While child welfare concerns may arise during a FEP, well‐developed social support services and strong links between EIP teams and child protection agencies could help women and families navigate these challenges, ensuring both maternal well‐being and child safety.

Clinicians consistently emphasised the need for trauma‐informed practice, noting that many women entering services had histories of sexual abuse or intimate‐partner violence. These findings resonate with broader evidence linking trauma exposure to psychosis risk and poorer engagement (Havig 2008; Ades et al. 2019). Participants observed that women often felt unsafe in male‐dominated clinical environments and that having access to female clinicians or women‐only spaces improved trust and disclosure, underscoring the relational dimension of safety in mental‐health care.

### 4.4 Medication and Clinical Dilemmas

Weight gain from antipsychotic medication was widely described as a major barrier to adherence, echoing evidence on pressures on women surrounding body image and self‐esteem (González‐Rodríguez et al. 2023). Clinicians described balancing the need for symptom control against women's concerns about physical changes that threaten self‐image. Some reported switching to less weight‐promoting medications or closely monitoring metabolic side effects, while others emphasised early, transparent dialogue that validates women's concerns and jointly weighs clinical efficacy against quality of life. This negotiation, central to recovery‐oriented practice, reflects a shared dilemma between therapeutic effectiveness and embodied wellbeing (Naughton et al. 2024).

### 4.5 Recommendations for Improving Care Included

#### 4.5.1 Enhanced Education and Training

Clinicians reported limited exposure to hormonal and gender‐related issues during their professional development. Embedding this knowledge within continuing education could improve assessment, prescribing, and awareness of sex–gender interactions (Mazza et al. 2021).

#### 4.5.2 Bidirectional Integrated Care

Participants described psychiatry, gynaecology, and endocrinology as operating in silos, linked only by one‐way referrals. Co‐located or networked models were recommended to enable shared decision‐making and real‐time consultation while maintaining women's autonomy in choosing their care providers (Barker and Vigod 2023).

#### 4.5.3 Trauma‐Informed, Sex and Gender‐Sensitive Environments

Clinicians advocated for gender‐matched practitioners, women‐specific groups, and trauma‐informed assessment. Dedicated women's units integrating psychiatric, reproductive, and social supports, successfully piloted elsewhere (González‐Rodríguez et al. 2023), were viewed as promising templates for EIP adaptation.

#### 4.5.4 Flexible and Socially Integrated Care

Flexibility in service hours, home visits, and co‐located childcare were identified as key to improving access. Family‐inclusive service models, such as women's health hubs that integrate psychiatry, primary care, childcare, and social supports (e.g., housing or domestic violence resources), were viewed as practical strategies to reduce barriers and sustain engagement while maintaining clinician wellbeing (Loveday et al. 2024; Jain et al. 2024; Radley et al. 2022).

### 4.6 Strengths and Limitations

This study intentionally foregrounds clinicians' perspectives to illuminate how sex‐ and gender‐responsive care is understood and enacted within EIP. While this lens offers service‐level insight, it does not capture the lived experiences of women or perspectives from adjacent disciplines (e.g., gynaecology, endocrinology, social care). Participants and services typically used binary, cis‐normative categories; consequently, the analysis may not represent trans and gender‐diverse experiences of psychosis or care, an explicit priority for future research and service design. The sample (*n* = 20) is appropriate for RTA, which emphasises depth over breadth; conducted within a single Irish EIP network (18–65), findings reflect this context; replication in settings with 18–35 limits and differing service architectures will clarify transferability.

## 5 Conclusion

This study highlights the unique and often overlooked challenges faced by cis‐gendered women with FEP, emphasising the need for sex‐ and gender‐responsive approaches to care and clinician education across disciplines. Clinicians recognise the critical role that hormonal influences, caregiving responsibilities, and stigma play in shaping women's experiences within EIP services. Future research should include service‐user perspectives and gender‐diverse populations to develop more inclusive models of care. To improve engagement and outcomes, mental health services must adopt flexible, integrated, and trauma‐informed approaches that address both biological and social determinants. By embedding biosocial awareness into practice, EIP services can better support all individuals affected by psychosis, regardless of gender identity or life stage.

## Funding

This research was funded by the St. John of God Research Foundation (2024_2037).

## Ethics Statement

This study was approved by the St. John of God Research Ethics Committee (ID2024‐2037). All procedures were conducted in accordance with the ethical standards of the responsible committee and with the 1964 Helsinki Declaration and its later amendments.

## Consent

Written informed consent was obtained from all participants involved in the study. Participants were provided with detailed information about the purpose, procedures, and confidentiality of the study prior to giving consent.

## Conflicts of Interest

The authors declare no conflicts of interest.

## Supporting information


**Data S1:** Reflexivity Statement.

## Data Availability

The data that support the findings of this study are available on request from the corresponding author. The data are not publicly available due to privacy or ethical restrictions.
