# Supplementary material for: Women's Care in First‐Episode Psychosis: Clinicians' Perspectives on Service Provision
Source: Early Interv Psychiatry. 2026 Apr 9;20(4):e70163. doi: 10.1111/eip.70163 (PMC13063352; doi:10.1111/eip.70163)
Supplement: Supplementary file 1 — Data S1: Reflexivity Statement. [file EIP-20-0-s001.docx]

Appendix A: Reflexivity Statement

As a 29-year-old female researcher exploring sex differences in first-episode psychosis (FEP), my positionality inevitably shapes my interpretation of the data. My experiences within the healthcare system both as a researcher and as a patient with endometriosis and psoriatic arthritis have made me acutely aware of the ways in which gender influences diagnosis, treatment, and healthcare interactions.

My own journey navigating chronic illness has exposed me to gendered patterns of medical treatment, where women’s symptoms are often minimized, attributed to stress, or misdiagnosed. This resonates with the findings in my research, particularly the potential longer duration of untreated psychosis (DUP) in women, the complexity of their presentations, and the way their symptoms may be overlooked or misattributed before reaching clinical recognition. It raises important questions for me: Are women’s psychotic symptoms, like their physical health concerns, dismissed or misunderstood? Does the tendency to see women’s presentations as more “complicated” reflect biological and social realities, or a healthcare bias that struggles to accommodate complexity?

My background also makes me particularly attuned to the interplay between biological, hormonal, and psychological factors. Living with endometriosis, I have firsthand experience of how hormonal fluctuations can affect mood, cognition, and well-being. This perspective influences my engagement with the data showing that women’s psychosis is often linked to perimenopause, postnatal changes, and emotional regulation difficulties. It leads me to critically reflect on whether the medical model of psychosis adequately considers the role of hormonal and autoimmune factors in women’s mental health, or if these aspects are overlooked.

Furthermore, my experience with chronic illness and medical gaslighting has made me deeply aware of how difficult it can be for women to have their symptoms taken seriously. The idea that women with psychosis may struggle to be convinced that they are unwell, as seen in my data, resonates with my own experiences of needing to advocate for my health. This makes me particularly reflective about the ways in which healthcare professionals perceive and engage with women’s mental health symptoms.

While my lived experience provides valuable insight, I recognize the need for ongoing reflexivity to ensure that my interpretations are grounded in the data rather than personal assumptions. My critical stance toward gender disparities in healthcare may make me more sensitive to patterns of systemic bias, but I must also ensure that I remain open to alternative explanations and diverse perspectives within the dataset. I acknowledge that not all women experience healthcare in the same way, and my own positionality, shaped by my specific health conditions and social context, may not reflect the full spectrum of female experiences in psychosis care.

Engaging with reflexive thematic analysis allows me to navigate these tensions, recognizing that my own position is both a strength and a potential source of bias. By remaining aware of my subjectivity, I aim to approach this research with both critical engagement and intellectual humility, ensuring that the voices of the participants remain central while also drawing on my unique insights as a researcher with lived experience.
